# Nutritional Ketosis and Mitohormesis: Potential Implications for Mitochondrial Function and Human Health

**DOI:** 10.1155/2018/5157645

**Published:** 2018-02-11

**Authors:** Vincent J. Miller, Frederick A. Villamena, Jeff S. Volek

**Affiliations:** ^1^Department of Human Sciences, College of Education and Human Ecology, The Ohio State University, Columbus, OH, USA; ^2^Department of Biological Chemistry and Pharmacology, College of Medicine, The Ohio State University, Columbus, OH, USA

## Abstract

Impaired mitochondrial function often results in excessive production of reactive oxygen species (ROS) and is involved in the etiology of many chronic diseases, including cardiovascular disease, diabetes, neurodegenerative disorders, and cancer. Moderate levels of mitochondrial ROS, however, can protect against chronic disease by inducing upregulation of mitochondrial capacity and endogenous antioxidant defense. This phenomenon, referred to as mitohormesis, is induced through increased reliance on mitochondrial respiration, which can occur through diet or exercise. Nutritional ketosis is a safe and physiological metabolic state induced through a ketogenic diet low in carbohydrate and moderate in protein. Such a diet increases reliance on mitochondrial respiration and may, therefore, induce mitohormesis. Furthermore, the ketone *β*-hydroxybutyrate (BHB), which is elevated during nutritional ketosis to levels no greater than those resulting from fasting, acts as a signaling molecule in addition to its traditionally known role as an energy substrate. BHB signaling induces adaptations similar to mitohormesis, thereby expanding the potential benefit of nutritional ketosis beyond carbohydrate restriction. This review describes the evidence supporting enhancement of mitochondrial function and endogenous antioxidant defense in response to nutritional ketosis, as well as the potential mechanisms leading to these adaptations.

## 1. Introduction

All cells of the human body require ATP as the fundamental energy source to support life. Because mitochondria produce the majority of ATP, impaired mitochondrial function is implicated in the majority of today's most concerning chronic and degenerative health conditions including obesity, cardiovascular disease, cancer, diabetes, sarcopenia, and neurodegenerative diseases [1]. Much of this association between mitochondrial function and disease can be attributed to excessive mitochondrial production of reactive oxygen species (ROS) [2].

Although mitochondrial ROS (mtROS) are generally considered harmful, which is certainly the case at high concentrations, modest levels stimulate necessary biological processes such as proliferation, differentiation, and immunity [3]. Adaptations that enhance resistance to oxidative stress are also induced by mtROS [3], possibly decreasing net ROS production during basal metabolism. This adaptive response is called mitohormesis [4–6] and is a promising mechanism through which lifestyle interventions that enhance mitochondrial function may, in turn, enhance resistance to chronic and degenerative diseases.

By dramatically shifting energy metabolism towards ketogenesis and fatty acid oxidation, ketogenic diets are likely to have a profound effect on mitochondrial function. However, despite the rapidly growing amount of research on ketogenic diets and their effects on various disease states, only a small amount of this research has focused on mitochondrial function or oxidative stress. The well-established increase in fat oxidation induced by a ketogenic diet [7, 8] clearly indicates prominent connection with mitochondrial function and, in turn, oxidative stress and mitohormesis [5, 6, 9]. Therefore, the purpose of this review is to describe the current, but limited, understanding of how ketogenic diets may affect mitochondrial function and resistance to oxidative stress, particularly within the context of extending human healthspan.

## 2. Nutritional Ketosis

The use of lifestyle interventions to treat and prevent chronic disease is attractive because of their potential to lower medical costs and produce more robust and holistic improvements in health. Ketogenic diets have been studied sporadically for more than 100 years, but over the last 15 years, a growing number of researchers have contributed to what is now a critical mass of discoveries that link the process of keto-adaptation to a broad range of health benefits [10–33]. Early clinical research focused on the use of “extreme” versions of ketogenic diets to treat seizures, but recent research indicates that benefits related to the management of epilepsy, weight loss, metabolic syndrome, and type 2 diabetes can be achieved with an approach that is less restrictive in carbohydrate and protein, and therefore more satisfying, sustainable, and feasible for the general population. A “well-formulated” ketogenic diet is generally characterized by a total carbohydrate intake of less than 50 g/d and a moderate protein intake of approximately 1.5 g/d per kg of reference weight [34]. This typically increases circulating *β*-hydroxybutyrate (BHB) and acetoacetate (ACA) from concentrations that are typically less than 0.3 mM into the range of nutritional ketosis, which for BHB, we define as 0.5–3 mM [35]. This range is below the typical 5–10 mM range for BHB that occurs during prolonged fasting, and well below concentrations characteristic of ketoacidosis [34, 35]. From the perspective of meeting energy demands, the reduced carbohydrate and moderate protein intakes necessarily make ketogenic diets high in fat. Despite this contradiction with mainstream dietary guidelines, ketogenic diets may be beneficial for many health conditions, particularly the previously mentioned conditions related to mitochondrial impairment, which includes obesity [10, 11], diabetes [12–14], cardiovascular disease [15–17], cancer [15, 18–26], neurodegenerative diseases [19, 20, 27–30], and even aging [31–33, 36, 37].

In both the nutrition literature and public dietary guidelines, nonstarchy vegetables are one of the few dietary components nearly unanimously agreed upon as healthful. Given their health-supporting characteristics and low carbohydrate content, they should be a prominent component of any ketogenic diet. Beyond the primary features of a well-formulated ketogenic diet, such as macronutrient proportions, adequate mineral intake, and appropriate selection of fat sources, which have been discussed more thoroughly elsewhere [34, 35], inclusion of nonstarchy vegetables is an important consideration, given that reports in the literature of adverse effects resulting from ketogenic diets are often associated with extreme implementations that typically lack plant matter. In fact, for this reason, it has recently been recommended to increase the nonstarchy vegetable content of ketogenic diets used to treat epilepsy [38]. Beyond adding variety to the diet, benefits of nonstarchy vegetables that may be particularly relevant to nutritional ketosis include the maintenance of adequate micronutrient status and the presence of prebiotic fiber as substrate for the gut microbiota. In addition to the importance of prebiotic fiber for basic health, the short-chain fatty acids produced by the gut microbiota from this dietary fiber support ketogenesis [39] and metabolic signaling related to mitochondrial function and antioxidant defense [40]. Furthermore, nonstarchy vegetables are a source of the many micronutrients needed to support energy metabolism. As such, there is more to a ketogenic diet than simply restricting carbohydrate. Selection of a variety of nutrient-dense foods is therefore an important component of nutritional ketosis that should be given consideration in any clinical or academic implementation.

## 3. Formation of mtROS and Associated Antioxidant Defense

As with other sources of oxidative stress, mtROS can damage enzymes and cell membranes and subsequently facilitate the pathogenesis of chronic disease [41]. Oxidative damage to mitochondrial DNA (mtDNA) is of particular concern because of its proximity to the electron transport chain (mtETC). Furthermore, compared to nuclear DNA, mtDNA is more prone to oxidative damage and is not repaired as effectively [42–45], although this has been debated based on more recent evidence [46–50]. Unrepaired mtDNA damage leads to mitochondrial dysfunction, which is implicated in the pathogenesis of a variety of chronic diseases [51] and associated with shorter lifespan [52]. Therefore, while moderate levels of mtROS have important roles in signaling and health, protection against excessive levels is also important.

Although there are numerous sites of mtROS formation, the most prominent are those in the mtETC, where the superoxide radical (O_2_^•−^) is formed through reduction of O_2_ by leaked electrons, particularly at complexes I and III [41, 53–55]. Production of O_2_^•−^ at complex I is particularly high during reverse electron transport (RET), which occurs when a high proton-motive force (Δ*p*) develops across the inner mitochondrial membrane in conjunction with the pool of coenzyme Q (CoQ) being in a highly reduced state (i.e., mostly present as ubiquinol) as a result of electron transfer through complex II and electron transfer flavoprotein:ubiquinone oxidoreductase (ETF-QO) [56–62]. This dependence of mtROS production on Δ*p* during RET is mainly influenced by proton gradient (ΔpH) [59].

Formation of O_2_^•−^ at complexes I and III primarily occurs in the mitochondrial matrix, but some of the O_2_^•−^ produced at complex III is produced in the intermembrane space [63]. Within the matrix, O_2_^•−^ is rapidly dismutated into hydrogen peroxide (H_2_O_2_) by manganese superoxide dismutase (SOD2) [41, 53]. Some O_2_^•−^ may escape into the mitochondrial intermembrane space [64] and cytosol [65], where copper/zinc superoxide dismutase (SOD1) can dismutate it into H_2_O_2_ [41]. The large majority of mitochondrial H_2_O_2_ is removed by peroxiredoxin (Prx) 3, followed by much smaller contributions from Prx5 and glutathione peroxidases (GPx) 1 and 4 [66]. GPx also removes other peroxides, including lipid hydroperoxides [41]. Catalase is another antioxidant enzyme capable of removing H_2_O_2_ but is primarily located in peroxisomes and is therefore unlikely to directly remove mitochondrial H_2_O_2_ [41, 66]. However, H_2_O_2_ can be transported out of mitochondria [67], and it is possible that the majority of mitochondrial H_2_O_2_ is removed in the cytosol. Since Prxs and GPxs rely on NADPH for recycling of their cofactors (thioredoxins and glutathione, resp.) [41], and since NADH is required for recycling of NADPH [68], activity of these enzymes would decrease availability of NADH for oxidative phosphorylation. Therefore, transport of H_2_O_2_ out of mitochondria for removal in the cytosol may be a more likely defense mechanism [67], implying a more important role of catalase and other antioxidant enzymes outside of mitochondria. Despite the lower reactivity of H_2_O_2_, it is still reactive and can oxidize metal ions, particularly iron, to form the hydroxyl radical (^•^OH), which readily damages DNA, lipids, and proteins [41]. ^•^OH is scavenged by metallothioneins I and II [69, 70] and glutatathione [71], indicating that these antioxidant proteins may be important defenses against byproducts of unaddressed mtROS. Other important antioxidant enzymes include glutamate-cysteine ligase (GCL), which is the rate-limiting step in glutathione synthesis, and glutathione reductase (GSR) and thioredoxin reductase (TRXR), which recycle glutathione and thioredoxin, respectively, to their reduced forms [41].

## 4. Mitohormesis

Increased reliance on mitochondrial respiration will increase the flow of electrons through the mtETC and, in turn, increase the potential for mtROS formation. Although oxidative stress is traditionally viewed as harmful, a modest increase in ROS is now established as a signaling stimulus that induces hormetic adaptation [3]. In regard to mitohormesis and mtROS, such adaptation is largely centered around antioxidant defense [4–6], making mitohormesis an attractive target for the prevention and treatment of chronic disease.

Although mitohormesis has not been studied comprehensively in higher-level organisms, its occurrence is supported by compelling evidence in lower-level organisms. For example, inhibition of glycolysis in *C. elegans* increased fat oxidation (based on nematode triglyceride content) and mitochondrial O_2_ consumption, which was followed by increases in ROS production at day 2 and catalase activity at day 6 [72]. The increase in catalase activity occurred in conjunction with increases in lifespan and resistance to the mitochondrial stressors sodium azide and paraquat. However, antioxidant treatment (*N*-acetylcysteine) decreased the elevation of ROS at day 2 and eliminated the resistance to sodium azide and paraquat treatments, indicating a requirement of ROS as a stimulus [72].

In a subsequent series of experiments, glucose metabolism in *C. elegans* was inhibited by knockdown of the insulin receptor, insulin-like growth factor 1 (IGF-1) receptor, and insulin receptor substrate 1 (IRS-1) [73]. Consistent with the previous study [72], inhibition of glucose metabolism increased mitochondrial respiration concomitant with ROS-dependent increases in lifespan, stress resistance, and antioxidant enzyme activity. However, in this case, detection of ROS was mitochondria-specific, and repeated measures allowed for changes in antioxidant enzyme activities to be evaluated more closely in relation to the timing of changes in mtROS. Compared to controls, inhibition of glucose metabolism resulted in higher mitochondrial O_2_ consumption at 12 h, higher mtROS production at 24 h, and higher activities of SOD and catalase at 48 h, suggesting a dependence of antioxidant activity on mtROS and a dependence of mtROS on mitochondrial respiration. The most striking result is the lower mtROS at 120 h, indicating that the initial increase in mtROS and subsequent increase in antioxidant enzyme activity ultimately lowered net mtROS production to a level lower than controls, which is the proposed explanation for the more than twofold increase in lifespan. As with the previous study, this demonstration of mitohormesis is further supported by the changes in ROS production, antioxidant enzyme activity, and lifespan having been prevented with antioxidant treatment.

The occurrence of mitohormesis is further supported by the potential for mtROS to simultaneously induce bioenergetic and antioxidant adaptations through a single signaling mediator. As discussed later in this review, this mediator is the transcription factor peroxisome proliferator-activated receptor *γ* coactivator 1*α* (PGC-1*α*), and its role in simultaneously inducing bioenergetic and antioxidant adaptations has been demonstrated in several experimental models of mtROS production, including treatments with paraquat and H_2_O_2_. Paraquat is an herbicide that is reduced by the mtETC and subsequently initiates mtROS production by reacting with O_2_ to produce O_2_^•−^ [74, 75], and H_2_O_2_ is a common form of mtROS. Treatment of a fibroblast cell line (10T1/2) with H_2_O_2_ has induced expression of both antioxidant enzymes (SOD1, SOD2, and GPx1) and proteins involved in mitochondrial oxidative phosphorylation, all in a manner largely dependent on PGC-1*α* [76]. Demonstrating the hormetic benefit of this response in a variety of brain cells, overexpression of PGC-1*α* protected against cell death induced by H_2_O_2_ or paraquat treatment, and this occurred in conjunction with changes in gene expression similar to those observed with the 10T1/2 cells [76]. Although the central role of PGC-1*α* in linking mitochondrial bioenergetics with antioxidant defense appears to not have been thoroughly investigated in vivo, some suggestive evidence does exist. In skeletal muscle of mice treated with paraquat, content of proteins involved in mitochondrial oxidative phosphorylation and uncoupling have been found to increase in conjunction with greater nuclear localization of PGC-1*α* [77]. Traditional antioxidant proteins were not measured, but, as will be discussed later, the increase in uncoupling proteins can be regarded as an indication of enhanced antioxidant defense based on the potential of these proteins to decrease mtROS production.

Ketogenic and low-carbohydrate diets greatly increase reliance on fat oxidation [78–89], which would logically be expected to increase mitochondrial respiration and mtROS production and, in turn, induce mitohormesis. Furthermore, mtROS produced through RET appears to have particular relevance to hormetic adaptation, including increased lifespan [90]. Nutritional ketosis is likely to increase RET by altering the FADH_2_ to NADH ratio. As the primary source of acetyl CoA shifts from glycolysis to *β*-oxidation and ketolysis, this ratio increases, more than doubling for *β*-oxidation of longer-chain fatty acids. Electrons from FADH_2_ reduce the CoQ pool through complex II and ETF-QO, thereby increasing RET [91, 92]. This induction of RET by alteration of substrate availability can also be influenced by configuration of mtETC complexes into supercomplexes [90]. The greater potential for mtROS production through RET is consistent with evidence of mitochondria producing more H_2_O_2_ during oxidation of palmitoyl carnitine versus pyruvate [93, 94]. Furthermore, succinate is generated during ketolysis by succinyl-CoA:3-oxoacid CoA-transferase (SCOT), which also promotes RET by reducing the CoQ pool through complex II. Demonstrating the likely role of RET in mitohormesis, particularly within the context of nutritional ketosis, extension of lifespan in *C. elegans* through BHB treatment is dependent on both complex I function and expression of bioenergetic and antioxidant proteins [95].

A study on hippocampal mitochondrial function in rats more directly supports the induction of mitohormesis by a ketogenic diet. After the first day of the diet (Bio-Serv F3666), H_2_O_2_ production by isolated mitochondria was increased [96]. After the third day, mitochondrial levels of oxidized glutathione (GSSG) and hippocampal levels of 4-hydroxy-2-nonenal (4-HNE) were also increased, further indicating an increase in oxidative stress. However, at completion of the first week, upregulation of antioxidant signaling occurred, indicated by increased nuclear content and transcriptional activity of nuclear factor erythroid-derived 2-like 2 (NFE2L2), which persisted through the remainder of the study. By the third week, mitochondrial H_2_O_2_ production decreased to below baseline [96]. In the liver, content of reduced acetyl CoA, which is indicative of mitochondrial redox status, decreased after three days of the ketogenic diet, but increased relative to the control diet after three weeks, indicating an initial increase in oxidative stress followed by a decrease [96]. This was in conjunction with changes in NFE2L2 nuclear content and transcriptional activity similar to those observed in the hippocampus. As with the previously described *C. elegans* experiments, the time course of these observations is a strong indication of mitohormesis, and the similarity in results between the liver and hippocampus suggests that a ketogenic diet can induce mitohormesis in a variety of tissues.

Several other rodent studies provide additional evidence of ketogenic diets upregulating antioxidant defense, but without enough data to convincingly attribute the results to mitohormesis. Content of SOD2 has increased in the livers of mice fed a ketogenic diet (% energy: 89 fat, <1 carbohydrate, and 10 protein), which occurred in conjunction with increased median lifespan and decreases in tumors and age-associated losses of physical and cognitive performance [36]. In addition, activity of GCL and the protein content of its two subunits increased in the hippocampal homogenate of rats fed a ketogenic diet (Bio-Serv F3666) for 3 weeks [97]. This was in conjunction with higher levels of reduced glutathione (GSH) and lower ROS production in hippocampal mitochondria. The ketogenic diet also increased resistance to mtDNA damage in hippocampal mitochondria exposed to H_2_O_2_ [97]. Consistent with these results, total antioxidant capacity and activities of GPx and catalase were increased in hippocampal homogenate of rats fed a ketogenic diet (% energy: 86 fat, <1 carbohydrate, and 13 protein) for 8 weeks [98]. Furthermore, in cortical homogenate of rats induced with traumatic brain injury, a ketogenic diet increased cytosolic and mitochondrial protein contents of NAD(P)H:quinone oxidoreductase 1 (NQO1) and SOD1, as well as mitochondrial protein content of SOD2, and also prevented mitochondrial oxidative damage (indicated by 4-HNE) [99].

Additional evidence, although disparate and primarily based on neuronal mitochondrial function related to epileptic seizures, further supports the potential for nutritional ketosis to induce mitohormesis [9]. Much of this is based on signal transduction, antioxidant defense, and oxidative capacity, all of which will be discussed in proceeding sections.

## 5. Ketones as Antioxidants and Signaling Molecules

**Figure 1:**
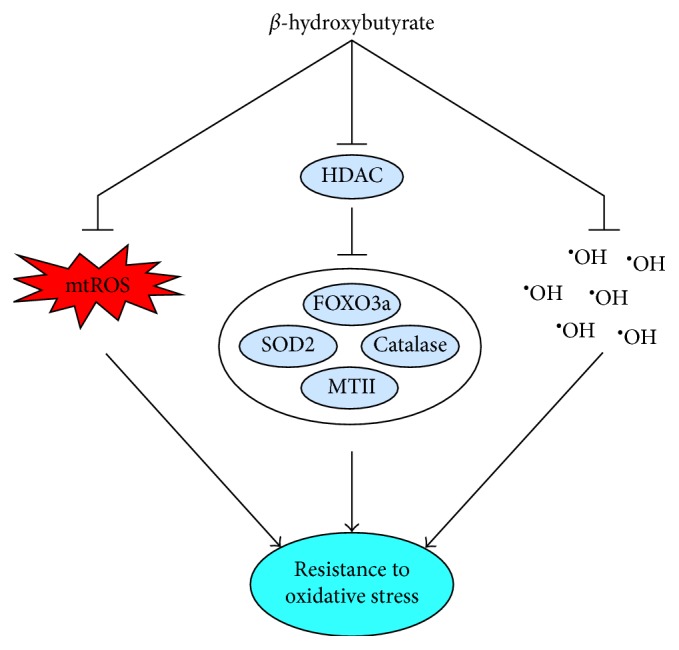
*β*-hydroxybutyrate and, in some cases, acetoacetate contribute to protection against oxidative stress by decreasing production of mitochondrial reactive oxygen species (mtROS), by increasing expression or protein content of antioxidant enzymes through histone deacetylase (HDAC) inhibition, and by directly scavenging the hydroxyl radical (^•^OH). Upregulation of antioxidant enzymes through HDAC inhibition includes manganese superoxide dismutase (SOD2), catalase, and metallothionein II and is likely mediated by the transcription factor forkhead box O 3a (FOXO3a).

Although ketones may not induce mitohormesis directly, they do influence antioxidant defense (Figure 1). Furthermore, ketone metabolism is highly relevant to mitochondrial adaptation since the ketogenic and ketolytic enzymes needed to support nutritional ketosis are located in mitochondria.

BHB, in addition to being an important energy substrate, is also a signaling molecule [100–102]. Although not induced through mtROS, BHB inhibits class I and II histone deacetylases (HDACs) in a dose-dependent manner, resulting in greater histone acetylation regardless of whether BHB is elevated through fasting, caloric restriction, or infusion [103]. This inhibition is associated with increased expression of forkhead box O (FOXO) 3a and metallothionein II and increased protein content of FOXO3a, SOD2, and catalase [103]. Consistent with these changes, the kidneys of mice with elevated blood BHB concentrations (∼1.2 mM) were protected from paraquat-induced (50 mg/kg) oxidative damage to proteins and lipids, which was indicated by lower levels of protein carbonyls, 4-HNE, and lipid peroxides [103]. Upregulation of antioxidant defense by BHB-induced HDAC inhibition also appears to be the mechanism through which exogenous BHB extends lifespan in *C. elegans* [95]. The dependence of this response on FOXO3a, NFE2L2, and several bioenergetic signaling proteins that influence the activities of these two transcription factors [95] is indicative of the overlap between bioenergetics and antioxidant defense that is characteristic of mitohormesis.

Additional indications of exogenous BHB upregulating antioxidant defense have been observed, although without consideration of HDAC inhibition. In rats, injection of BHB has increased activities of SOD and catalase and prevented the increase in lipid peroxidation and decreases in SOD, catalase, and GSH induced by paraquat injection, all of which were observed in kidney homogenate [104]. Furthermore, BHB also prevented the paraquat-induced decrease in nuclear NFE2L2, indicating involvement of antioxidant signaling [104]. Similarly, BHB treatment has increased FOXO3a, SOD2, and catalase content in cardiomyocytes [105], indicating that BHB may also influence antioxidant defense in the heart. In this study, BHB also prevented the decrease of FOXO3a, SOD2, and catalase content that resulted from H_2_O_2_ treatment [105]. Despite the amount of research that has been done on the antiseizure mechanisms of ketogenic diets, the influence of BHB on HDAC inhibition and related antioxidant defense appears to have not yet been investigated in brain tissue. However, BHB appears to inhibit HDAC2 in microvascular and neuronal brain cells [106], and BHB-induced HDAC inhibition is thought to have a role in the antiseizure effects of ketogenic diets [107].

In addition to BHB inducing upregulation of antioxidant defense, ketones have direct antioxidant capacity. BHB scavenges ^•^OH, as does ACA, although to a lesser extent [108]. The applicability of this antioxidant capacity has been investigated in vitro and in vivo in the context of hypoglycemia. In cultured hippocampal neurons, treatment with BHB or ACA decreased ROS during hypoglycemia induced through inhibition of glycolysis, and in hypoglycemic rats, infusion of BHB decreased hippocampal lipid peroxidation [108].

More specific to mitochondrial function, treatment with BHB + ACA (1 mM each) has decreased O_2_^•−^ production in isolated rat neuronal mitochondria following glutamate exposure [109]. This occurred in conjunction with decreased NADH levels, suggesting that ketones may additionally decrease mtROS production by enhancing electron transport along the mtETC after NADH oxidation and, in turn, decreasing mitochondrial Δ*p* and associated O_2_^•−^ production. The observed decrease in mitochondrial O_2_^•−^ production occurred independently of glutathione [109], but in isolated and stunned hearts from guinea pigs, treatment with 5 mM ACA increased GSH and the NADPH/NADP^+^ ratio [110], suggesting that glutathione may be involved to some extent.

Further indicating that ketones influence mtROS production through alteration of electron transport, treatment of rat hippocampal slices with BHB + ACA (1 mM each) prevented the increase in mtROS and mitigated the decrease in ATP production that otherwise result from inhibition of mtETC complex I with rotenone [111]. In mitochondria isolated from the brains of mice injected with BHB, although inhibition of complex I with rotenone and 1-methyl-4-phenylpyridinium increased rather than decreased mtROS production, the BHB treatment prevented the decrease in O_2_ consumption caused by inhibition of complex I, and this occurred independently of uncoupling [112]. Consistent with the results from hippocampal brain slices, the BHB treatment also mitigated the decrease in ATP production caused by complex I inhibition [112]. These effects were prevented by inhibition of complex II with 3-nitropropionic acid or malonate, indicating that BHB primarily influences mitochondrial respiration at complex II [112], which is consistent with ketolysis increasing formation of succinate and FADH_2_. However, in mutated cells prone to complex I disassembly and an associated severe decrease in complex I activity, treatment with BHB + ACA (5 mM each) increased both the assembly and activity of complex I [113], indicating that ketones somehow promote repair of complex I damage and may therefore influence mitochondrial respiration at more than one site.

Another aspect of mitochondrial function influenced by ketones is the mitochondrial permeability transition pore (mPTP). Prolonged opening of the mPTP is one of the mechanisms through which mtROS can induce cellular injury and promote disease [114]. In neurons isolated from rat brain slices, treatment with BHB + ACA has decreased the mtROS production, mPTP opening, and cell death induced by H_2_O_2_ [115]. This protective effect was duplicated with catalase, even in conjunction with diamide-induced opening of the mPTP, indicating that the protective effect of BHB and ACA is at least partly due to defense against ROS [115]. In a mouse model of epilepsy, this decrease in mPTP opening was found to be induced exclusively by BHB, and in a manner dependent on the cyclophilin D subunit of the mPTP [116]. BHB in combination with ACA also appears to promote opening of mitochondrial ATP-sensitive K^+^ (mtK_ATP_) channels [117], which in heart mitochondria is known to protect against Ca^+^ overload [118] and dissipate membrane potential (Δ*Ψ*) [119]. Since high Δ*Ψ* promotes mtROS production, dissipation of Δ*Ψ* through mtK_ATP_ channels may partly explain the potential for ketones to decrease mtROS production. However, opening of mtK_ATP_ channels by pinacidil decreases mitochondrial ATP production [119], which is consistent with dissipation of Δ*Ψ* and suggests a compromise between ATP and mtROS production.

Ketones may also be important, or even necessary, for the bioenergetic signaling associated with mitohormesis. As will be discussed later, peroxisome proliferator-activated receptor *α* (PPAR*α*) is a nuclear receptor that is responsible for many of the bioenergetic adaptations associated with nutritional ketosis and mitohormesis [120]. In mice, a ketogenic diet (% energy: 90 fat, 0 carbohydrate, and 10 protein) increased blood BHB concentration to 1-2 mM and upregulated expression of numerous PPAR*α* targets in the liver [37]. However, in mice fed a nonketogenic low-carbohydrate diet (% energy: 75 fat, 15 carbohydrate, and 10 protein), which did not raise blood concentration of BHB, the increased expression of PPAR*α* targets did not occur [37], implying that induction of PPAR*α* signaling by a ketogenic diet is dependent on ketones. This response may be, at least in part, a result of the epigenetic effects of BHB. In addition to HDAC inhibition, BHB also influences gene expression through *β*-hydroxybutyrylation of histone lysine residues [121]. In the livers of mice subjected to prolonged fasting, this *β*-hydroxybutyrylation has been associated with upregulation of PPAR signaling, oxidative phosphorylation, fatty acid metabolism, the proteasome, and amino acid metabolism related to redox balance [121]. Upregulation of these pathways is largely influenced by *β*-hydroxybutyrylation of the histone residue H3K9 [121], which is also involved in the upregulation of antioxidant defense through BHB-induced HDAC inhibition [103]. This potential for BHB to influence expression of both mitochondrial and antioxidant genes through a common histone residue is further indication of the overlap between bioenergetics and antioxidant defense and suggests that if mitohormesis is indeed induced during nutritional ketosis, induction may be dependent on ketones and may therefore not occur during a low-carbohydrate diet that is not ketogenic.

In regard to the practicality of BHB signaling, many of the outcomes described above, including HDAC inhibition, were achieved with BHB concentrations within the range of 0.6–2 mM [37, 103, 105, 108, 109, 111, 112, 116], which is well within the physiological range of nutritional ketosis for humans and even suggests potential benefit at low to moderate levels.

## 6. Mitochondrial Uncoupling

As previously discussed, RET is a prominent source of mtROS and is dependent on a high Δ*p* across the inner mitochondrial membrane. During ATP production, Δ*p* is dissipated as H^+^ enters the mitochondrial matrix through ATP synthase. Mitochondrial uncoupling also dissipates Δ*p*, but by allowing translocation of H^+^ into the matrix independent of ATP synthase. Uncoupling is therefore regarded as an antioxidant defense in that it can mitigate mtROS production [122–126]. In fact, only a small dissipation of Δ*Ψ* or ΔpH (components of Δ*p*) is needed for a large decrease in mtROS production [57–60, 127].

Mitochondrial uncoupling is primarily facilitated by uncoupling proteins (UCPs) and adenine nucleotide translocase (ANT) [124, 128, 129]. Although UCP1 is primarily expressed in brown adipose, UCP2 is expressed across a wide variety of tissues, and expression of UCP3 appears to be limited to skeletal muscle and the heart [130]. Knockout of UCP2 [131] or UCP3 [94, 132] increases mtROS production, and both proteins are inactivated through glutathionylation by GSH [133], further establishing their involvement in antioxidant defense. UCP2 and UCP3 may also be activated by products of lipid peroxidation induced by mtROS [122]. However, the potential for UCP2 and UCP3 to reduce mtROS through uncoupling is not fully agreed upon; [128] UCPs may alternatively protect against oxidative damage merely by exporting lipid hydroperoxides [128]. Furthermore, UCP3 is less abundant in type I and type IIa muscle fibers [134], which are more oxidative, and its expression and content are further decreased by endurance exercise training [135, 136], suggesting that UCP3 may not be a primary defense against mtROS.

Although the primary purpose of ANT is to exchange newly synthesized ATP in the mitochondrial matrix for cytosolic ADP [129], it shares a common feature with UCPs and other inner membrane proteins in that they translocate anions, including fatty acids. The uncoupling mechanism of ANT, along with UCP2 and UCP3, may be the exchange of protonated fatty acids from the mitochondrial intermembrane space for fatty acid anions in the matrix, thereby dissipating Δ*p* [123, 137–139]. Inhibition studies indicate that ANT may contribute more to uncoupling than UCPs [140, 141].

Independent of nutritional ketosis, increased dietary fat intake increases expression of UCP2 and UCP3 in muscle [142], and fatty acids facilitate uncoupling through UCP2 [143, 144], UCP3 [94, 143, 144], and ANT [145]. Given the high fat intake that is characteristic of a ketogenic diet, it is logical to expect nutritional ketosis to increase mitochondrial uncoupling.

Certain ionophores are capable of completely uncoupling mitochondria by transporting H^+^ across the inner membrane. Such ionophores are therefore commonly used to measure maximal mitochondrial respiration. In mice fed a ketogenic diet (Bio-Serv F3666, ∼6  :  1 ratio of fat to carbohydrate + protein) for 6 days, respiration of hippocampal mitochondria was fully uncoupled with the ionophore carbonyl cyanide 4-(trifluoromethoxy)phenylhydrazone (FCCP) [146]. The ratio of respiration during oxidation of palmitic acid to maximally uncoupled respiration induced by FCCP was greater in response to the ketogenic diet, indicating increased uncoupling [146]. Although this interpretation relies on the assumption that ATP production was not changed by diet, it is further supported by the higher levels of UCP2, UCP4, and UCP5 detected in mitochondria after the ketogenic diet. Furthermore, mtROS production was lower in the ketogenic diet group [146], supporting the role of uncoupling as an antioxidant defense. Although not based on direct measurement of mitochondrial function, in rats fed a ketogenic diet (% energy: 89.5 fat, 0.1 carbohydrate, and 10.4 protein), increased uncoupling in response to nutritional ketosis is further indicated by increases in fat oxidation and overall O_2_ consumption occurring in conjunction with decreases in CO_2_ production and energy expenditure [89]. However, based on observations of greater palmitate-induced uncoupling (determined by measurement of Δ*Ψ*) during state 4 respiration in rats fed a high-fat, low carbohydrate diet (% energy: 50 fat, 21 carbohydrate, and 29 protein) [147] that was likely too high in carbohydrate and protein to induce nutritional ketosis, it is possible that moderate carbohydrate restriction may increase mitochondrial uncoupling independently of ketones.

Several additional rodent studies have shown ketogenic diets to increase protein content of UCPs. However, since mitochondrial function was not measured in these studies, it is not known if uncoupling was affected by these changes in UCP content. In obese mice fed a ketogenic diet (0.4% of energy as carbohydrate), expression of UCP1 and UCP2 increased in adipose and the liver, respectively [148]. Similarly, expression of UCP1 has increased in brown adipose of mice fed a low-carbohydrate diet (18.5% of energy) supplemented with ketone esters (6% *w*/*v*) [149]. The increase in hepatic UCP2 expression during a ketogenic diet has been demonstrated by other studies as well [37, 150, 151]. Ketogenic diets also induce expression of UCP3 in skeletal muscle. In rats fed a ketogenic diet (% energy: 78.1 fat, 0 carbohydrate, and 21.9 protein) for 8 weeks, UCP3 expression increased in the soleus but not the extensor digitorum longus, which is consistent with the soleus containing mostly oxidative, type I muscle fibers [152]. In humans, glycogen depleting exercise followed by two days of a low-carbohydrate diet (0.7 g/kg body mass) increased UCP3 expression in the vastus lateralis [153].

## 7. Oxidative Capacity

As the rate of oxidative phosphorylation approaches the capacity of the mtETC, Δ*p* will increase and facilitate mtROS production [53]. Higher oxidative capacity should therefore decrease the potential for mtROS production and subsequent oxidative damage. Furthermore, greater oxidative capacity may compensate for the resulting decrease in efficiency of ATP production associated with increased mitochondrial uncoupling. Since oxidative phosphorylation occurs exclusively in mitochondria, mitochondrial density is a key determinant of oxidative capacity [154].

As previously mentioned, numerous studies have demonstrated a profound increase in fat oxidation in response to ketogenic and low-carbohydrate diets. Some studies have even shown an increase in O_2_ consumption [148, 155–158]. However, fats contain fewer oxygen atoms per carbon than carbohydrates, thereby necessitating greater O_2_ intake to produce the same amount of energy [159]. Furthermore, since *β*-oxidation and ketolysis produce a greater proportion of FADH_2_ to NADH, the resulting decrease in passage of electrons through complex I decreases potential for ATP production per unit of O_2_ consumption [160]. Increased O_2_ consumption in response to a ketogenic diet may therefore merely be an effect of the differences in the metabolism and molecular structures of fat and carbohydrate rather than a true indication of increased capacity for oxidative phosphorylation. However, in rat hearts perfused with glucose, the addition of ketones has decreased O_2_ consumption [161]. This discrepancy may be related to variations in mitochondrial uncoupling. Either way, several studies have shown ketogenic diets to increase mitochondrial content, and numerous studies have shown these diets to increase expression, content, or activity of mitochondrial proteins involved in oxidative phosphorylation and fat oxidation. Compared to O_2_ consumption alone, these findings provide more conclusive support for an increase in oxidative capacity in response to nutritional ketosis.

In rats fed a ketogenic diet (Bio-Serv F3666) for 22 days, mitochondrial density (determined by electron microscopy) in the hippocampus increased in conjunction with increased transcription of 39 of the 42 mitochondrial proteins analyzed [162]. Similarly, mitochondrial content (mtDNA copy number) increased in skeletal muscle of mice fed a ketogenic diet (Research Diets D05052004; % energy: 89.5 fat, 0.1 carbohydrate, and 10.4 protein) for 10 months [163]. Higher mtDNA copy number was also observed in skeletal muscle of rats fed a high-fat, low-carbohydrate diet (% energy: 60 fat, 20 carbohydrate, and 20 protein) for 4 weeks in conjunction with daily injections of heparin (0.5 U/g) to increase circulation of fatty acids [87]. In humans, after just 3 days of a low-carbohydrate, high-fat diet (% energy: 50 fat, 34 carbohydrate, and 16 protein), fat oxidation significantly increased and 49% of the variance was explained by mtDNA content [79]. Despite this, the content of mtDNA did not change significantly, but this was expected given the brief duration of the diet.

As will be discussed in the following sections, many of the signaling proteins involved in regulating antioxidant defense also regulate oxidative phosphorylation and fat oxidation. There is abundant evidence (Table 1) showing ketogenic and low-carbohydrate diets to increase expression, content, or activity of many targets of these signaling proteins, further indicating increased oxidative capacity. It is particularly striking that ketogenic or low-carbohydrate diets upregulate expression of proteins associated with each of the five mtETC complexes.

## 8. Bioenergetic Signal Transduction

Perturbations in bioenergetic homeostasis induce signal transduction that leads to upregulation of mitochondrial capacity and antioxidant defense. Three key enzymes involved in the sensing of these perturbations and the subsequent induction of signal transduction are AMP-activated protein kinase (AMPK) and silent mating type information regulation 2 homologues 1 and 3 (SIRT1 and SIRT3).

### 8.1. AMPK

AMPK is activated through phosphorylation of the Thr^172^ residue of the AMPK *α* catalytic subunit [174–176], and this phosphorylation is largely regulated by molecules related to bioenergetic homeostasis including AMP, ADP, catecholamines, adiponectin, glycogen, and insulin. In general, AMPK is activated by energy deficit and induces signaling that upregulates energy production. AMP and ADP are direct byproducts of energy depletion while adiponectin and catecholamines serve as endocrine signals to increase energy production, often in response to energy depletion. In contrast, indications of energy surplus, such as glycogen and insulin, inhibit activation of AMPK. Nutritional ketosis increases the aforementioned factors that activate AMPK and decreases those that inhibit AMPK, suggesting that nutritional ketosis is similar to caloric restriction in inducing a signal of energy depletion.

AMP competes with ATP for binding to the *γ* regulatory subunit of AMPK [177, 178] and by doing so, greatly increases AMPK activity, but only in the presence of an upstream kinase such as liver kinase B1 (LKB1) [179]. This binding of AMP to the *γ* subunit appears to promote AMPK activity through at least two mechanisms: facilitated phosphorylation of the *α* subunit [180–183] and inhibition of dephosphorylation by protein phosphatases 2C*α* and 2Ac [179, 181, 183, 184]. ADP also binds to the *γ* subunit of AMPK to inhibit dephosphorylation [183, 185, 186] and possibly facilitate phosphorylation [185]. This is important to the energy sensing sensitivity of AMPK based on the much higher physiological concentration of ADP compared to AMP [186]. Data on changes in AMP and ADP levels in response to a ketogenic diet are lacking. However, the decreased availability of carbohydrate and increased mitochondrial uncoupling (previously described) during nutritional ketosis suggest a decline in ATP production, at least until compensatory adaptations occur. A decline in ATP implies a relative increase in AMP and ADP, which would facilitate AMPK phosphorylation and activation. In addition, ketogenic diets are commonly reported to have a satiating effect [187], which may further increase the AMP and ADP to ATP ratios through spontaneous caloric restriction.

Adiponectin increases AMPK activity in skeletal muscle [188, 189] and the liver [189] by promoting Thr^172^ phosphorylation, likely in response to an increase in the AMP to ATP ratio [189]. Similarly, *α*-adrenergic signaling increases AMPK activity in skeletal [190] and cardiac muscle [191], and *β*-adrenergic signaling increases AMPK activity in adipose [192, 193], all through promotion of Thr^172^ phosphorylation. While activation through *β*-adrenergic signaling appears to involve the AMP to ATP ratio [192], *α*-adrenergic signaling appears to work independently of AMP and ATP [190]. Increases in adiponectin have been observed during ketogenic or low-carbohydrate diets, although primarily in obese individuals [194–196]. BHB induces adiponectin secretion in adipocytes [197], indicating that the level of nutritional ketosis may be an important determinant of the extent to which ketogenic diets influence AMPK activity through adiponectin. In regard to catecholamines, epinephrine increases during fasting, and this appears to be dependent on carbohydrate restriction [198], implying that epinephrine is likely to be elevated during nutritional ketosis. Consistent with this, dietary carbohydrate restriction increases catecholamines at rest [155, 199] and in response to exercise [155, 199–202]. This may be, at least in part, a result of glycogen depletion [200, 203], suggesting both direct and indirect effects of glycogen on AMPK activity. The potential for nutritional ketosis to increase catecholamines is further supported by the dependency of the antiseizure effects of ketogenic diets on norepinephrine [204].

Glycogen influences AMPK activity by binding to a glycogen binding domain on the *β* regulatory subunit of AMPK [205, 206]. In human and rodent skeletal muscle, AMPK activity is lower when glycogen is bound to this domain [207, 208] and higher when muscle is depleted of glycogen [209–212]. In direct contrast to the effect of AMP and ADP, glycogen inhibits the phosphorylation of AMPK by upstream kinases such as LKB1 [213]. Although muscle glycogen concentration has recently been demonstrated to be similar in ultra-endurance athletes regardless of a ketogenic or high-carbohydrate diet [8], concentrations generally decrease in response to dietary carbohydrate restriction [156, 166, 173, 214–221]. Furthermore, the long-term adaptations to nutritional ketosis that may enable some athletes to replenish glycogen at a normal rate may not apply to less physically active individuals.

Insulin inhibits AMPK activity by stimulating protein kinase B (PKB) to phosphorylate the Ser^485^ residue of the *α* subunit, thereby inhibiting phosphorylation at Thr^172^ [222]. One of the most prominent features of nutritional ketosis is that, due to restricted carbohydrate intake, postprandial insulin is dramatically decreased. Furthermore, numerous studies have shown ketogenic or low-carbohydrate diets to decrease fasting insulin [155, 195, 223–225], particularly in the presence of metabolic dysregulation associated with hyperinsulinemia [84, 226–229].

Consistent with the mechanisms described above, changes in AMPK in response to a ketogenic or low-carbohydrate diet have been reported in several studies. In rodents, a ketogenic diet (Bio-Serv F3666) has increased AMPK activity in skeletal muscle [150] and AMPK phosphorylation in the liver [230], and a low-carbohydrate diet (18.5% of energy) supplemented with ketone esters (6% *w*/*v*) increased AMPK content in brown adipose [149]. In humans, a nonketogenic low-carbohydrate diet (% energy: 50 fat, 30 carbohydrate, and 20 protein) has increased AMPK phosphorylation in skeletal muscle [231].

### 8.2. SIRT1 and SIRT3

The sirtuin isoforms SIRT1 [232, 233] and SIRT3 [234–236] are nicotinamide adenine dinucleotide- (NAD^+^) dependent deacetylases associated with longevity. Many reactions are regulated by the redox state of NAD^+^ and its phosphorylated form, NADP^+^. Among these reactions, a prominent role of reduced NADP^+^ (i.e., NADPH) is to support reductive biosynthesis and antioxidant defense, requiring the NADP^+^/NADPH ratio to be kept low [237]. In contrast, the NAD^+^/NADH ratio is kept high to support energy metabolism [237], thereby linking sirtuin function to bioenergetic status [238]. Although sirtuins are inhibited by high concentrations of NADH, their activity is influenced more by absolute NAD^+^ concentration than the NAD^+^/NADH ratio [238].

SIRT1 is present in the cytosol and nucleus [239], while SIRT3 is primarily located in mitochondria where it regulates bioenergetics and ROS production [239–241]. The sirtuins, particularly SIRT1, appear to participate in a feed-forward cycle of reciprocal activation with AMPK. In skeletal muscle, AMPK indirectly activates SIRT1 by increasing NAD^+^ through increased mitochondrial *β*-oxidation [242] and increased expression of nicotinamide phosphoribosyltransferase (NAMPT) [243], which is the rate-limiting enzyme in NAD^+^ synthesis [244]. Completing the cycle, SIRT1 and SIRT3 can deacetylate and activate LKB1, thereby promoting further activation of AMPK. LKB1 is known to be activated by SIRT1 in adipose and liver [245] and by SIRT3 in cardiac muscle [246].

Based on the reciprocal activation described above, nutritional ketosis is likely to activate SIRT1 and SIRT3 indirectly through activation of AMPK. However, more direct activation of sirtuins by nutritional ketosis is possible. Since reduction of NAD^+^ to NADH occurs outside of mitochondria only during glycolysis, which is less active during nutritional ketosis, more cytosolic NAD^+^ remains oxidized, further facilitating activation of SIRT1 [247]. In addition to the decrease in glucose availability during nutritional ketosis, glycolysis may be further inhibited through activation of pyruvate dehydrogenase kinase and subsequent inhibition of pyruvate dehydrogenase (PDH), which occurs in response to dietary carbohydrate restriction [248–251] or infusion of BHB, ACA, or fatty acids [252]. Consistent with the relevance of these factors to nutritional ketosis, a ketogenic diet (% energy: 89 fat, <1 carbohydrate, and 10 protein) has decreased expression of PDH in mouse liver [36]. More importantly, there is direct evidence of nutritional ketosis promoting an increase in NAD^+^ concentration. Treatment with BHB + ACA (1 mM each) has increased NADH oxidation in rat neocortical mitochondria [109], and a ketogenic diet (Bio-Serv F3666) has increased NAD^+^ concentration in rat hippocampus [253]. There is also evidence of nutritional ketosis regulating sirtuin expression. A low-carbohydrate (20% of energy) diet combined with ketone esters (6% *w*/*v*) has increased SIRT1 protein content in brown adipose of mice [149], and a ketogenic diet (% energy: 90 fat, 0 carbohydrate, and 10 protein) has increased SIRT3 expression in mouse liver [37].

In addition to the downstream bioenergetic and antioxidant signaling induced by sirtuins, they directly facilitate ketogenesis and *β*-oxidation. SIRT1 [254] and SIRT3 [255] deacetylate 3-hydroxy-3-methylglutaryl CoA (HMG CoA) synthase, which is the rate-limiting enzyme for ketogenesis [256], resulting in increased levels of *β*-hydroxybutyrate [255]. In addition, SIRT3 deacetylates and increases activity of long-chain acyl-CoA dehydrogenase (LCAD) [257], which participates in *β*-oxidation and therefore supports ketogenesis. SIRT3 has a similar influence on medium-chain acyl-CoA dehydrogenase (MCAD) as well [258]. Since sirtuins facilitate ketogenesis, which then facilitates sirtuin activation, nutritional ketosis may promote, to some extent, a feed-forward cycle of sirtuin activity.

### 8.3. Direct Involvement of AMPK and Sirtuins in Redox Balance

Although the majority of links between energy sensing and antioxidant defense are manifested further downstream, there is some direct influence at the level of AMPK and sirtuins. AMPK is activated by oxidative stress [259, 260], likely through ATP depletion and a subsequent increase in the AMP to ATP ratio, or facilitation of tyrosine phosphorylation, which occurs independently of AMP and ATP concentrations [259]. SIRT3 contributes more directly to antioxidant defense by deacetylating and activating SOD2 [261–263]. The overlapping effect of SIRT3 on antioxidant defense and bioenergetics is further supported by SIRT3 knockout increasing lipid peroxidation in conjunction with decreased O_2_ consumption in mouse skeletal muscle and also by SIRT3 knockdown increasing H_2_O_2_ production and decreasing O_2_ consumption in myoblasts [264].

## 9. Downstream Bioenergetic and Antioxidant Signaling

AMPK and sirtuins are the interface between the metabolic stimuli of nutritional ketosis and the downstream signaling that influences expression of proteins related to bioenergetics and antioxidant defense. Some of the primary downstream signaling molecules involved include PGC-1*α*, FOXO3a, nuclear respiratory factors 1 and 2 (NRF-1 and NRF-2), mitochondrial transcription factor A (TFAM), and NFE2L2.

### 9.1. PGC-1α

The coordinated effects of AMPK, SIRT1, and SIRT3 are primarily mediated through PGC-1*α*, which is activated through phosphorylation by AMPK [242, 265] and deacetylation by SIRT1 [77, 242, 266–269]. SIRT3 also increases PGC-1*α* activity [270], possibly through cAMP response element binding protein (CREB) [271, 272], but the exact mechanism has not been elucidated. In addition to phosphorylating PGC-1*α*, activated AMPK also increases PGC-1*α* expression [260, 273–276]. Activation of *β*_2_-adrenergic receptors [277–280] and the adiponectin AdipoR1 receptor [281] also increase PGC-1*α* expression, independently of AMPK activation [278, 281]. PGC-1*α* activity is increased by oxidative stress [76, 77, 282–284], possibly through activation of AMPK [259, 260] or p38 mitogen-activated protein kinase (MAPK) [283, 284], or inhibition of glycogen synthase kinase 3*β*, which inhibits PGC-1*α* through phosphorylation [77, 283]. In contrast, insulin decreases PGC-1*α* activity through phosphorylation by PKB [285]. Once activated, PGC-1*α* interacts with the PPAR family of nuclear receptors [286] and the FOXO family of transcription factors [287] to influence expression of a variety of bioenergetic and antioxidant proteins. PGC-1*α* most notably increases transcription of proteins involved in mitochondrial biogenesis and respiration [76, 242, 265, 267, 269, 274, 279, 282, 285, 288–293] but also increases transcription of antioxidant proteins including SOD1 [76], SOD2 [76, 282, 289, 292–294], catalase [282], GPx [76, 294], thioredoxins [282, 283, 292], TRXR [282, 292], Prx3 [282, 292], and Prx5 [282, 292], as well as the mitochondrial uncoupling proteins UCP2 [76, 265, 282, 288, 294], UCP3 [76, 265, 294], and ANT [76, 295].

PGC-1*α* coactivates all three known PPAR isoforms (PPAR*α*, PPAR*δ*, and PPAR*γ*) [286]. Although each isoform is expressed in a variety of tissues, PPAR*α* is prominently expressed in the liver, PPAR*δ* in skeletal muscle, the heart, and the pancreas, and PPAR*γ* in adipose [286, 296]. PGC-1*α* was discovered and named based on its promotion of brown adipose differentiation through coactivation of PPAR*γ* and subsequent induction of mitochondrial biogenesis and UCP1 expression [297]. However, it is the PGC-1*α* coactivation of PPAR*α* that is responsible for the upregulated transcription of many of the enzymes responsible for increased ketogenesis and fatty acid metabolism in response to a ketogenic diet [120]. Consistent with the role of PGC-1*α* in inducing mitochondrial biogenesis, it also shifts skeletal muscle fiber composition towards type I [298, 299] and type IIa [299], which are more oxidative. AMPK also contributes to fiber type changes and is required for the transition of highly glycolytic, type IIb fibers to more oxidative, type IIa fibers [276]. Although PGC-1*α* is primarily known for inducing transcription of nuclear DNA, it may also, in conjunction with SIRT1, induce expression of mtDNA [300].

PGC-1*α* is also influenced by p38 MAPK, which is well known for being involved in development [301] and adaptation [302] in skeletal muscle. PGC-1*α* is activated by p38 MAPK [283, 303] through phosphorylation [304], which prevents repression [303] by blocking interaction with the p160 myb binding protein [304]. In addition, expression of PGC-1*α* is increased by p38 MAPK [305, 306], and the overlap in bioenergetic and antioxidant signaling is further indicated based on p38 MAPK activation by AMPK [307–309], oxidative stress [310–314], and *β*-adrenergic signaling [280, 315, 316].

Nutritional ketosis may facilitate PGC-1*α* activity through multiple mechanisms. Since PGC-1*α* is activated by AMPK and SIRT1, nutritional ketosis may initiate PGC-1*α* activity through these enzymes. As previously mentioned, catecholamines and adiponectin facilitate PGC-1*α* activity by promoting its expression, and insulin inhibits PGC-1*α* through downstream phosphorylation, all independent of AMPK. As previously discussed, a ketogenic diet may increase catecholamines and adiponectin and is well known to decrease insulin, indicating that nutritional ketosis may directly facilitate PGC-1*α* activity through these hormones. Supporting these potential mechanisms, a ketogenic or low-carbohydrate diet has increased expression, protein content, and activation of PGC-1*α* [149, 231, 317], as well as expression of its target PPAR*α* [87, 148]. Furthermore, in skeletal muscle of mice following a ketogenic diet, the resulting increases in O_2_ consumption and expression of genes related to fat oxidation appear to be dependent on PGC-1*α* [157]. Ketones likely contribute to this signaling as well based on the recent observation that the increased hepatic expression of PPAR*α* targets induced by a ketogenic diet did not occur with a nonketogenic low-carbohydrate diet [37].

### 9.2. FOXO3a

The FOXO family of transcription factors is highly conserved and promotes longevity and resistance to cellular stress. Although there are a variety of FOXO isoforms with varying tissue distribution [318–320], FOXO3a has been the most thoroughly studied in relation to energy sensing, mitochondrial function, and antioxidant defense. Similar to PGC-1*α*, FOXO3a is activated through phosphorylation by AMPK [321–323] and deacetylation by SIRT1 [324, 325] and SIRT3 [326–329], and its transcriptional activity is at least partly dependent on AMPK [322] and SIRT1 [325]. In a variety of organisms, tissues, and cell types, FOXO3a increases mitochondrial biogenesis and expression of TFAM [329], but is more known for increasing expression of antioxidant and repair proteins, including SOD2 [287, 330, 331], catalase [287, 330, 332, 333], glutathione S-transferase (GST) [322], thioredoxins [287, 323], Prx3 [287, 334], Prx5 [287], and metallothioneins I and II [322], as well as UCP2 [287, 322] and the DNA repair enzyme growth arrest and DNA damage-inducible 45 (GADD45) [322, 324, 335, 336]. FOXO3a is also activated by oxidative stress [324, 331, 333], possibly in a SIRT1-dependent manner [324], and likely mediated through c-Jun N-terminal protein kinase (JNK), which allows FOXOs to translocate to the nucleus by promoting dissociation of 14-3-3 [337, 338]. Furthermore, FOXO3a and SIRT3 interact in mitochondria to induce mitochondrial gene expression in an AMPK-dependent manner [339]. FOXO3a also induces expression of LKB1 [340] and NAMPT [341], indicating a feed-forward cycle of activation with AMPK and sirtuins. Like PGC-1*α*, FOXO3a transcriptional activity is inhibited by insulin through PKB [331].

As with PGC-1*α*, nutritional ketosis may activate FOXO3a by increasing activity of AMPK and sirtuins or by decreasing insulin. Expression of FOXO3a is increased by fasting, caloric restriction, and BHB [103, 105], all of which are or can be components of a ketogenic diet. Furthermore, BHB treatment has extended lifespan in *C. elegans* in a manner dependent on FOXO3a [95], and a ketogenic diet (% energy: 89 fat, <1 carbohydrate, and 10 protein) has increased median lifespan and decreased tumors and age-associated losses of physical and cognitive performance, all in conjunction with increased hepatic content of FOXO3a [36].

### 9.3. NRF-1, NRF-2, and TFAM

NRF-1 and NRF-2 are transcription factors that increase expression of TFAM [342], which is required for full initiation of mtDNA transcription [343–345] and hence mitochondrial biogenesis. PGC-1*α* induces expression of NRF-1 and NRF-2 and facilitates TFAM expression by coactivating NRF-1 [288]. Oxidative stress increases this signaling [346, 347] in conjunction with increased mitochondrial biogenesis [346]. AMPK also contributes to mitochondrial biogenesis, but by inducing mitochondrial fission through phosphorylation of mitochondrial fission factor (MFF) [348], which is in addition to and independent of AMPK's role in activating PGC-1*α*.

### 9.4. NFE2L2

Nuclear factor erythroid-derived 2-like 2 (NFE2L2 or NRF2) is a transcription factor that has a prominent role in antioxidant signaling and also influences mitochondrial bioenergetics. The NFE2L2 abbreviation is used in this review to avoid confusion with nuclear respiratory factor 2, which despite being a different protein, has overlapping function with NFE2L2 and shares the same NRF2 abbreviation [349]. Although the mechanisms of NFE2L2 signaling are not fully elucidated [350], oxidative stress has a clear role in interacting with cysteine residues of Kelch-like ECH-associated protein 1 (Keap1), which decreases proteasomal degradation of NFE2L2 and thereby allows entry of NFE2L2 into the nucleus to induce transcription [351–355]. Although the influence of PGC-1*α* on antioxidant enzyme expression is not dependent on NFE2L2 [76, 356], PGC-1*α* increases NFE2L2 expression [357], indicating that NFE2L2 activity is influenced by perturbations in both energy and redox homeostasis. NFE2L2 primarily increases expression of antioxidant enzymes, including SOD1 [358], SOD2 [358], catalase [358–361], GPx [360], NQO1 [354, 359–362], GCL [359–361], GST [362], GSR [359–361], and Prx1 [352], but also increases expression of proteins involved in mitochondrial biogenesis and bioenergetics including NRF-1, NRF-2, TFAM, cytochrome c oxidase, and citrate synthase [358].

In the previously described *C. elegans* experiments demonstrating mitohormesis, knockout of the NFE2L2 homologue SKN-1 attenuated the increases in antioxidant enzyme activity and lifespan [73], indicating that mitohormesis may, at least in part, be dependent on NFE2L2 signaling. Similarly, a ketogenic diet (Bio-Serv F3666) increased nuclear content of NFE2L2 and expression of its target NQO1 in the hippocampi of rats, all of which occurred after an initial increase in mtROS [96]. This increase in NFE2L2 content appears to have mediated the subsequent decrease in mtROS to a level below baseline [96], thereby further indicating a likely role of NFE2L2 in the induction of mitohormesis during a ketogenic diet.

Additional evidence, although independent of mitohormesis, further supports the induction of NFE2L2 activity by nutritional ketosis. Succinate is a byproduct of ketolysis and is oxidized to fumarate by succinate dehydrogenase. Therefore, the increased presence of ketones and increased rate of ketolysis during nutritional ketosis are likely to increase fumarate, which can succinylate cysteine residues of proteins [363]. In particular, fumarate can succinylate Keap1, thereby allowing NFE2L2 to enter the nucleus to induce transcription [364, 365]. In the retinas of rats injected with BHB, the nuclear content of NFE2L2 and the total homogenate content of SOD2 and GCL increased in conjunction with increased fumarate concentration [366]. BHB injection also decreased retinal ROS production and degeneration following induction of ischemia, and this protection was dependent on NFE2L2 [366]. These effects were observed at blood concentrations of BHB between 1 and 2 mM, which is consistent with nutritional ketosis.

## 10. Overlap between Bioenergetic and Antioxidant Signal Transduction

**Figure 2:**
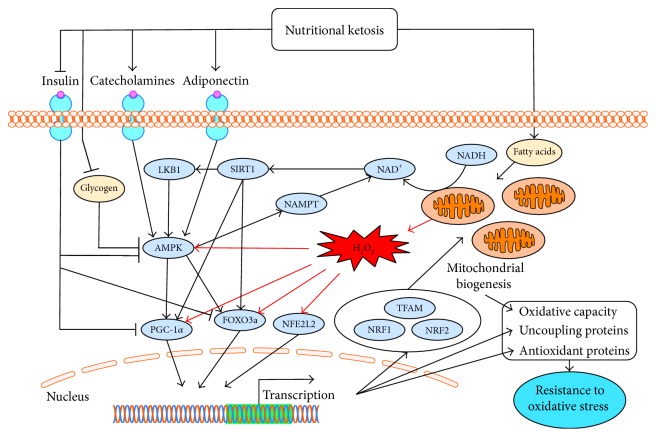
Nutritional ketosis may initiate bioenergetic and mitohormetic signaling through an increase in catecholamines or adiponectin, a decrease in insulin or glycogen, or an increase in *β*-oxidation that leads to an increase in mitochondrial reactive oxygen species (mtROS) or NAD^+^. This leads to further signaling involving AMP-activated protein kinase (AMPK), silent mating type information regulation 2 homologue 1 (SIRT1), peroxisome proliferator-activated receptor *γ* coactivator 1*α* (PGC-1*α*), forkhead box O 3a (FOXO3a), and nuclear factor erythroid-derived 2-like 2 (NFE2L2), ultimately leading to transcription of genes related to oxidative capacity, mitochondrial uncoupling, and antioxidant defense. These adaptations collectively contribute to resistance against oxidative stress. Other proteins involved include liver kinase B1 (LKB1), which activates AMPK; nicotinamide phosphoribosyltransferase (NAMPT), which facilitates SIRT1 activation through NAD^+^ synthesis; and nuclear respiratory factors 1 and 2 (NRF-1 and NRF-2) and mitochondrial transcription factor A (TFAM), which promote mitochondrial biogenesis.

As described throughout the previous sections, there are many instances of codependencies and feed-forward loops in bioenergetic and antioxidant signal transduction, which supports the well-known potential for metabolic stimuli, such as diet or exercise, to have a profound physiological influence. Given the central role of mitochondria in oxidative phosphorylation and ROS production, the overlap between bioenergetic and antioxidant signaling is not surprising and is possibly an outcome of evolution favoring efficiency. PGC-1*α* is at the center of this overlapping and complex network of codependencies. The likely role of PGC-1*α* as a coactivator of FOXO3a indicates a possible dependence of FOXO3a transcriptional activity on PGC-1*α* [287], indicating FOXO3a as a central mediator as well. Furthermore, FOXO3a induces transcription of PGC-1*α* [287, 322, 367], and formation and antioxidant transcriptional activity of the PGC-1*α*-FOXO3a complex are partly dependent on interaction with SIRT1 [325]. In muscle, expression of many of the bioenergetic and antioxidant proteins previously discussed is dependent on PGC-1*α* [265]. Upstream, activation of PGC-1*α* is dependent on AMPK [242] and SIRT1 [242, 269] and partly dependent on SIRT3 [270]. Furthermore, activation of SIRT1 is dependent on AMPK [242], which may also be the case for SIRT3. AMPK and PGC-1*α* are therefore two key factors, with critical supporting roles of the sirtuins, in the signal transduction linking bioenergetics to antioxidant defense. Further supporting the relevance of this linkage to nutritional ketosis, expression of AMPK, SIRT1, FOXO3a, and NFE2L2 is required for extension of lifespan in *C. elegans* by exogenous BHB [95], and expression of AMPK, p38 MAPK, and NFE2L2 is required for the extension of lifespan, also in *C. elegans*, by mitohormesis induced through inhibition of glucose metabolism [73]. The induction of AMPK [259, 260], SIRT3 [263, 329], p38 MAPK [310–313], PGC-1*α* [76, 77, 260, 282, 283], FOXO3a [324, 331, 333], and NFE2L2 [358–360] activity by oxidative stress also makes this signaling highly relevant to mitohormesis [263, 282, 360], especially given that activation of these proteins has been shown to decrease mitochondrial or cellular ROS [76, 263, 282, 289, 323, 332, 334, 356, 359, 367]. Furthermore, mitochondrial biogenesis [346] and the activities of AMPK [259, 260], SIRT3 [329], p38 MAPK [311, 312], PGC-1*α* [76, 77, 260, 283], FOXO3a [324], and NFE2L2 [368] are increased by H_2_O_2_, more specifically associating this signaling with mitohormesis. Given that AMPK and sirtuins are upstream of the majority of this signaling and that AMPK and sirtuin activities are stimulated by both bioenergetic and oxidative stressors, these stressors are likely the primary signals through which nutritional ketosis may induce the mitochondrial and antioxidant adaptations characteristic of mitohormesis (Figure 2).

## 11. Exercise as an Adjunct to Nutritional Ketosis

Although resting skeletal muscle is less metabolically active than the heart, kidneys, brain, or liver, it rivals even the brain in being the body's most metabolically demanding tissue when considered relative to total tissue mass [369]. Physical activity can greatly increase this demand, making exercise a practical and powerful way to induce bioenergetic adaptations.

In skeletal muscle, impaired mitochondrial function contributes to age-associated atrophy, impaired contraction, and insulin resistance [2]. While exercise provides a direct stimulus for mitochondrial adaptation in muscle, with great potential to prevent or treat the aforementioned conditions, the global effects of exercise on bioenergetic homeostasis may lead to mitochondrial adaptations in other tissues as well. Based on this, exercise has the potential to influence any condition for which impairments in global energy metabolism or local mitochondrial function are a contributing factor, which is arguably the case for a majority of chronic diseases. Exercise is therefore an excellent adjunct to nutritional ketosis because it facilitates *β*-oxidation and ketogenesis by increasing energy demand and depleting glycogen storage, which is likely to augment the signaling induced by nutritional ketosis.

In skeletal muscle, oxidative capacity and mitochondrial content are related to fiber type. Compared to type II fibers, type I fibers have larger mitochondria [370] with greater oxidative enzyme content [371]. While fiber type is plastic, particularly in response to endurance exercise, transformation from oxidative, slow-twitch fibers (type I) to glycolytic, fast-twitch fibers (type II) is unlikely to occur [372, 373]. Type II fibers, however, can shift in humans from highly glycolytic (type IIx) to more oxidative (type IIa) [373]. Compared to type IIx fibers, type IIa fibers have greater citrate synthase activity, indicating greater mitochondrial content [374]. The relevance of oxidative capacity and fiber type to oxidative stress has been demonstrated by greater mitochondrial respiration with less H_2_O_2_ production in permeabilized fibers from rat muscle consisting primarily of type I or IIa fibers versus type IIb fibers [375]. Although muscle fiber-type transformation has been well characterized in response to exercise, this appears to not be the case for ketogenic diets. However, in rats, *β*-hydroxyacyl-CoA dehydrogenase (*β*-HAD) has been shown to increase most prominently in glycolytic, type IIb fibers following 4 weeks of a ketogenic diet (% energy: 70 fat, 6 carbohydrate, and 24 protein) [165], suggesting transition of these fibers towards type IIa fibers and, in turn, indicating potential for nutritional ketosis to promote a more oxidative muscle fiber composition.

Bioenergetic and oxidative stressors may be largely responsible for inducing many of the beneficial adaptations to exercise, and for this reason, exercise research provides much of the basis for mitohormesis [4–6]. As previously discussed, an increase in fat oxidation appears to be a prerequisite for increasing mtROS and, in turn, inducing mitohormesis. Given that ketogenic diets prominently increase fat oxidation during submaximal exercise [8, 88, 214–216, 218, 219, 376–381], the combination of the two interventions may induce mitohormetic adaptations to a greater extent. Furthermore, much of the signaling that is relevant to mitohormesis, and likely induced by nutritional ketosis, is also induced by exercise, further suggesting the possibility of an additive or even synergistic effect. Demonstrating this, exercise or muscle contraction increases activity, activation, or expression of AMPK [209–211, 275, 284, 382–386], SIRT1 [384–389], SIRT3 [272, 390, 391], NFE2L2 [358, 360, 392], p38 MAPK [284, 305, 313–315, 393–395], PGC-1*α* [275–279, 284, 305, 314, 385–389, 396–400], NRF-1 [358], and TFAM [358, 388, 389]. Exercise also increases expression or activity of antioxidant enzymes [313, 358, 360, 396, 397, 401], uncoupling proteins [94], and bioenergetic proteins involved in oxidative phosphorylation [396, 397, 400] and the citric acid cycle [396], all of which appear to be at least partly mediated by ROS-induced activity of p38 MAPK [284, 310, 313, 314], PGC-1*α* [284, 310, 397, 401], TFAM [310, 314, 358, 397], NRF-1 [310, 358, 397], NRF-2 [358, 360], and NFE2L2 [358].

In addition to increased mitochondrial demand and mtROS production, there are several other commonalities in the mechanisms through which exercise and nutritional ketosis induce adaptive signaling. Exercise-induced activation of AMPK is greater when the exercise is performed in a glycogen depleted state [209–211, 382, 383], and exercise-induced activation of p38 MAPK [315] and PGC-1*α* [277–279] occurs at least partly through *β*-adrenergic signaling. Although changes in NAD^+^ and NADH are difficult to measure and are complicated by conflicting results, exercise is also likely to increase sirtuin activation by increasing the NAD^+^ to NADH ratio [402].

In controlled studies on exercise-trained humans and animals, ketogenic diets have been shown to increase fat oxidation [8, 167] and expression or activity of carnitine palmitoyltransferase [167, 168] and *β*-HAD [168, 172], demonstrating that nutritional ketosis induces adaptation beyond exercise. Similarly, in a study comparing the independent and combined effects of exercise and a ketogenic diet on rats, the combination resulted in greater *β*-HAD and citrate synthase activities in skeletal muscle and higher maximal O_2_ consumption than either intervention alone, further indicating the potential for exercise to magnify adaptations induced by nutritional ketosis [156].

## 12. Conclusion

Among the chronic and degenerative diseases in which impaired mitochondrial function is a contributing factor, many respond favorably to lifestyle interventions focused on diet and exercise. The therapeutic potential of nutritional ketosis stands out in this regard. For example, in just the first 10 weeks of an ongoing clinical trial with hundreds of type 2 diabetics following a ketogenic diet, glycated hemoglobin (HbA_1c_) decreased to below the diagnostic threshold in more than a third of patients, and prescription medication was reduced or eliminated for more than half of patients [12]. Convincing arguments for a ketogenic diet to be the default treatment for diabetes are a decade old [13] and have continued to gain support since then [14]. Similar arguments are developing for obesity [10, 11], neurodegenerative diseases [19, 20, 27–30], cardiovascular disease [15–17], cancer [18–26], and even aging [31, 32]. Although the mechanisms through which a ketogenic diet may improve these conditions expand beyond mitochondrial function, the great extent to which nutritional ketosis increases reliance on mitochondrial metabolism strongly suggests that mitochondrial adaptation is a central factor.

The clinical relevance of nutritional ketosis to mitochondrial function is further indicated by promotion of ketogenic diets for treatment of mitochondrial disorders [19, 20, 26, 30, 247, 403]. The most prominent example is the study of mitochondrial adaptations as a mechanism for the well-known antiseizure effect of ketogenic diets [19, 29, 33, 162, 247, 403–405]. As previously discussed, the dramatic shift in energy metabolism and subsequent increase in circulating ketones induced by a ketogenic diet can enhance mitochondrial function and endogenous antioxidant defense. The primary mechanism behind these adaptations appears to be the increased demand for fat oxidation resulting from carbohydrate restriction. However, ketones themselves have important metabolic and signaling effects that enhance mitochondrial function and endogenous antioxidant defense, implying that a well-formulated ketogenic diet should have greater benefit than a nonketogenic low-carbohydrate diet. Regardless of the mechanism(s), the potential outcomes imply protection against chronic disease through improved mitochondrial function and, in turn, decreased potential for oxidative stress and subsequent pathology. However, further research is needed to better understand how nutritional ketosis influences mitochondrial function across different tissues and how these influences relate to human disease. Future research should also focus on further differentiation of the effects of carbohydrate restriction from the direct effects of ketones.

## Figures and Tables

**Table 1:** Bioenergetic proteins upregulated by ketogenic or low-carbohydrate diets.

*Oxidative phosphorylation*	
NADH dehydrogenase (complex I)	[162]
Succinate dehydrogenase (complex II)	[87, 149, 162, 164, 165]
Cytochrome c reductase (complex III)	[162]
Cytochrome c oxidase (complex IV)	[87, 149, 162]
ATP synthase (complex V)	[87, 162]
Cytochrome c	[149, 162]
*Citric acid cycle*	
Citrate synthase	[156, 166]
Isocitrate dehydrogenase	[162]
Succinate dehydrogenase (complex II)	[87, 149, 162, 164, 165]
Malate dehydrogenase	[162, 165]
*Fatty acid oxidation*	
Carnitine palmitoyltransferase	[36, 37, 87, 152, 167–169]
Medium-chain acyl-CoA dehydrogenase (MCAD)	[36, 87, 148, 170]
Long-chain acyl-CoA dehydrogenase (LCAD)	[87, 148, 151]
Very-long-chain acyl-CoA dehydrogenase (VLCAD)	[87]
*β*-Hydroxyacyl-CoA dehydrogenase (*β*-HAD)	[148, 150–152, 156, 165, 166, 168, 169, 171–173]
*Ketolysis*	
*β*-Hydroxybutyrate dehydrogenase	[148, 150, 151]
